# Surveillance for *Ixodes scapularis* and *Ixodes pacificus* ticks and their associated pathogens in Canada, 2019

**DOI:** 10.14745/ccdr.v48i05a04

**Published:** 2022-05-05

**Authors:** Christy H Wilson, Salima Gasmi, Annie-Claude Bourgeois, Jacqueline Badcock, Navdeep Chahil, Manisha A Kulkarni, Min-Kuang Lee, L Robbin Lindsay, Patrick A Leighton, Muhammad G Morshed, Christa Smolarchuk, Jules K Koffi

**Affiliations:** 1 Centre for Food-borne, Environmental and Zoonotic Infectious Diseases, Public Health Agency of Canada, Ottawa, ON; 2 Centre for Food-borne, Environmental and Zoonotic Infectious Diseases, Public Health Agency of Canada, Saint-Hyacinthe, QC; 3 Public Health New Brunswick, New Brunswick Department of Health, Fredericton, NB; 4 BCCDC Public Health Laboratory, BC Centre for Disease Control, Vancouver, BC; 5 School of Epidemiology and Public Health, University of Ottawa, Ottawa, ON; 6 One Health Section, National Microbiology Laboratory Branch, Public Health Agency of Canada, Winnipeg, MB; 7 Epidemiology of Zoonoses and Public Health Research Group (GREZOSP), Faculty of Veterinary Medicine, Université de Montréal, Saint-Hyacinthe, QC; 8 Department of Pathology and Laboratory Medicine, University of British Columbia, Vancouver, BC; 9 Analytics and Performance Reporting Branch, Health Standards, Quality and Performance Division, Alberta Health, Edmonton, AB

**Keywords:** *Ixodes scapularis*, *Ixodes pacificus*, surveillance, Borrelia, Anaplasma, Babesia

## Abstract

**Background:**

The primary vectors of the agent of Lyme disease in Canada are *Ixodes scapularis* and *Ixodes pacificus* ticks. Surveillance for ticks and the pathogens they can transmit can inform local tick-borne disease risk and guide public health interventions. The objective of this article is to characterize passive and active surveillance of the main Lyme disease tick vectors in Canada in 2019 and the tick-borne pathogens they carry.

**Methods:**

Passive surveillance data were compiled from the National Microbiology Laboratory Branch and provincial public health data sources. Active surveillance was conducted in selected sentinel sites in all provinces. Descriptive analysis of ticks submitted and infection prevalence of tick-borne pathogens are presented. Seasonal and spatial trends are also described.

**Results:**

In passive surveillance, specimens of *I. scapularis* (n=9,858) were submitted from all provinces except British Columbia and *I. pacificus* (n=691) were submitted in British Columbia and Alberta. No ticks were submitted from the territories. The seasonal distribution pattern was bimodal for *I. scapularis* adults, but unimodal for *I. pacificus* adults. *Borrelia burgdorferi* was the most prevalent pathogen in *I. scapularis* (18.8%) and *I. pacificus* (0.3%). In active surveillance, *B. burgdorferi* was identified in 26.2% of *I. scapularis*; *Anaplasma phagocytophilum* in 3.4% of *I. scapularis*, and *Borrelia miyamotoi* and Powassan virus in 0.5% or fewer of *I. scapularis*. These same tick-borne pathogens were not found in the small number of *I. pacificus* tested.

**Conclusion:**

This surveillance article provides a snapshot of the main Lyme disease vectors in Canada and their associated pathogens, which can be used to monitor emerging risk areas for exposure to tick-borne pathogens.

## Introduction

*Ixodes scapularis* and *Ixodes pacificus* are tick vectors capable of transmitting several bacterial, viral and protozoan pathogens to humans ( ( ((1)))). *Ixodes scapularis* populations are increasing in number and distribution in southern central and eastern Canada ( ( ((2–4)))). Climate (e.g. increasing temperatures, changes in precipitation) and environmental factors (e.g. changes in land use) contribute to the geographic range expansion of ticks, which can enhance exposure to tick-borne diseases (TBD) ( ( ((1,5–7)))). These changes can also create longer seasons for adventitious ticks to become established in new areas and increase human-tick interactions ( ( ((1,4,6–8)))). Continued expansion of the range of ticks in Canada presents a public health challenge, as awareness of TBD risks and capacity for surveillance and testing must also expand to these areas ( ( ((1)))).Lyme disease (LD) is the most commonly reported vector-borne disease in Canada, and incidence of reported cases has increased more than 17-fold from 2009 through 2019 ( ( ((9,10)))). The causative agent of LD, *Borrelia burgdorferi*, is transmitted by *I. scapularis* in central and eastern Canada and by *I. pacificus* in British Columbia. Beyond LD, other TBD including anaplasmosis (caused by the bacterium *Anaplasma phagocytophilum*), babesiosis (caused by the parasite *Babesia microti*), hard tick-borne relapsing fever (caused by the bacterium *Borrelia miyamotoi*) and Powassan virus (POWV) disease are emerging as diseases locally acquired within Canada ( ( ((1,11–15)))).Passive surveillance began in the early 1990s in Canada to detect the occurrence of *I. scapularis* and *I. pacificus* tick vectors and their infection with *B. burgdorferi* ( ( ((16)))). Active surveillance has been ongoing since the 2000s to identify areas where vector tick populations are establishing and, as a result, where LD may become endemic (LD risk areas) ( ( ((17,18)))). This is the first edition of a pan-Canadian annual article summarizing the findings of both passive and active vector surveillance and updating estimates of infection prevalence in ticks. A previous study by Guillot *et al.* ( ( ((19)))) summarized results of a pan-Canadian study on tick surveillance; however, that study only included active tick surveillance from sentinel sites.The objective of this surveillance article is to provide an epidemiologic summary of the main LD vectors in Canada, *I. scapularis* and *I. pacificus*, and their associated pathogens, collected through active and passive surveillance systems in 2019. This article will also summarize the prevalence and spatial distribution of tick-borne pathogens.

## Methods

### Data sources

This article uses two types of surveillance data from six different sources: 1) passive tick surveillance data from the National Microbiology Laboratory (NML) Branch of the Public Health Agency of Canada, the British Columbia Centre for Disease Control and Alberta Health ( ( ((20)))); and 2) active tick surveillance data from the Canadian Lyme Sentinel Network (CaLSeN), the New Brunswick Department of Health and the University of Ottawa.

#### Passive tick surveillance

In passive tick surveillance, ticks are collected by the public and submitted to medical clinics, veterinary clinics, or directly to a provincial public health laboratory or other institution (e.g. university laboratory) for species identification ( ( ((16)))). Location of acquisition, history of travel in the past two weeks, date of collection, level of engorgement, tick instar and host are recorded.This article focuses on *I. scapularis* and *I. pacificus* ticks collected in Canada, although several other tick species were also collected. Ticks with an international location of acquisition, an imprecise location within Canada that could not be geocoded (e.g. province listed only, multiple locations listed) or history of travel were excluded to create a dataset of locally acquired ticks. Over the years, passive tick surveillance programs have been discontinued in different jurisdictions, i.e. Nova Scotia, southwestern Québec (Montérégie) and eastern Ontario; however, the public continues to submit a relatively small number of ticks acquired in these jurisdictions directly to NML.In 2019, Saskatchewan, Manitoba, Ontario, Québec, Newfoundland and Labrador, New Brunswick, Nova Scotia and Prince Edward Island sent ticks to NML for testing of tick-borne pathogens (*A. phagocytophilum*, *B. burgdorferi* and *B. microti*) using methods described previously ( ( ((21,22)))). Ticks could be submitted singly or in groups of two or more (multiple submission). For laboratory testing, ticks from the same multiple submission were pooled and tested together. In British Columbia ( ( ((23)))) and Alberta ( ( ((24)))), testing was done at provincially funded laboratories on individual ticks for only *B. burgdorferi*. Ticks are rarely encountered in northern Canada and as a result, formal passive tick surveillance programs for *I. scapularis* or *I. pacificus* are not established in the Yukon, Northwest Territories or Nunavut.

#### Active tick surveillance

Active surveillance involves collection of ticks in the environment through drag sampling or through capture of host mammals that are examined for ticks. This method aims to identify where emerging tick populations are establishing ( ( ((4,18)))). For this article, only *I. scapularis* and *I. pacificus* collected by drag sampling were included for analysis, although several other tick species were also collected.

This article collates data from CaLSeN, the New Brunswick Department of Health and the University of Ottawa. The CaLSeN used standardized methods to conduct dragging in 96 sites across all provinces ( ( ((19)))). The New Brunswick Department of Health and the University of Ottawa used similar dragging methods to visit 73 and 15 sites, respectively ( ( ((25)))). Visit date, location of collection (latitude and longitude), tick species and tick instar were recorded for all ticks collected.

Nymphs and adult *I. scapularis* and *I. pacificus* were tested for tick-borne pathogens. Ticks collected by CaLSeN and by the province of New Brunswick were tested for *A. phagocytophilum*, *B. microti*, *B. burgdorferi*, *B. miyamotoi* and POWV (CaLSeN ticks only) at NML using methods previously described ( ( ((19,21,22)))). Ticks collected by the University of Ottawa were tested for *A. phagocytophilum*, *B. burgdorferi* and *B. miyamotoi* with quantitative polymerase chain reaction (qPCR) assays described previously ( ( ((25)))) using the *flaB* gene for *B. miyamotoi* and including a confirmatory assay targeting *msp2* in *A. phagocytophilum*. Testing for *B. microti* used a qPCR assay targeted towards the *cctη* gene ( ( ((21)))).

### Analysis

#### Tick characteristics

For passive tick surveillance, we calculated descriptive statistics for province of acquisition, tick species, instar (larva, nymph, adult male or adult female), level of engorgement (unfed, partially engorged or fully engorged), host (human, dog, cat or other) and month of collection. For active tick surveillance, we calculated descriptive statistics for province of acquisition, tick species and instar (larva, nymph or adult). The probable location of acquisition for ticks was mapped using QGIS (version 3.8.1).

#### Infection prevalence

For ticks submitted through passive surveillance, maximum likelihood estimates (MLE) of prevalence with 95% confidence intervals (CI) were calculated in Excel (version 16.0) using the PooledInfRate add-in (version 4.0) to account for pooled testing ( ( ((26,27)))). Co-infection prevalence was assessed among single submissions only to ensure they were true co-infections (two or more pathogens in the same tick). The prevalence of co-infections was calculated as the number of co-infected ticks divided by the total number of ticks tested. Prevalence in active surveillance was calculated in the same manner as all ticks were tested individually.

## Results

### Passive surveillance tick characteristics

**Figure 1:**
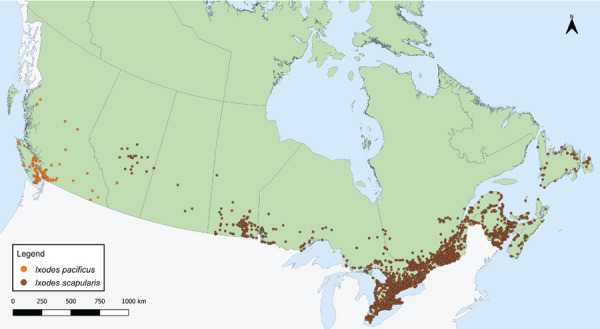
*Ixodes pacificus* and *Ixodes scapularis* ticks submitted through passive tick surveillance, Canada, 2019^a,b^ ^a^ Each dot represents the probable location of acquisition for an *I. pacificus* (n=691) or *I. scapularis* (n=9,858) tick submitted through passive surveillance ^b^ No passive surveillance was conducted in Yukon, Northwest Territories or Nunavut for *I. scapularis* or *I. pacificus* ticks. Passive tick surveillance has been discontinued in the entire province of Nova Scotia, and some regions of Ontario and Québec; however, individuals could submit ticks directly to the National Microbiology Laboratory Branch from these jurisdictions

In 2019, there were 10,549 *I. pacificus* and *I. scapularis* ticks submitted from all provinces through passive surveillance (**Table 1**). The majority of ticks (90.0%) were submitted from three provinces: Ontario, Québec, and New Brunswick (**Figure 1**). The majority of ticks (94.0%) were single submissions, but there were 242 multiple submissions (range: 2–8 ticks). Nova Scotia had the highest proportion of multiple submissions (13.7%; n=7/51).

**Table 1:** Number of *Ixodes scapularis* and *Ixodes pacificus* ticks and submissions collected through passive surveillance by province, Canada, 2019^a^

Province	Number of ticks			Number of single submissions^b^	Multiple submissions^b^		
	*Ixodes scapularis*	*Ixodes pacificus*	Total		Number of submissions	Median number of ticks per submission	
						n	Range
British Columbia	0	690	690	690	N/A^c^	N/A^c^	N/A^c^
Alberta	55	1	56	56	N/A^c^	N/A^c^	N/A^c^
Saskatchewan	3	0	3	3	0	N/A	N/A
Manitoba	175	0	175	149	8	3	2–7
Ontario^d^	6,857	0	6,857	6,436	167	2	2–8
Québec^d^	1,697	0	1,697	1,618	31	2	2–7
Newfoundland and Labrador	44	0	44	42	1	2	2
New Brunswick	941	0	941	868	28	2	2–8
Nova Scotia^e^	72	0	72	44	7	5	2–5
Prince Edward Island	14	0	14	14	0	N/A	N/A
Total	9,858	691	10,549	9,920	242	2	2–8

Abbreviation: N/A, not applicable

^a^ No passive surveillance was conducted in Yukon, Northwest Territories or Nunavut for *I. scapularis* or *I. pacificus* ticks

^b^ Single submissions consist of one tick; multiple submissions consist of two or more ticks submitted together by the same person

^c^ Province did not report whether ticks were from single or multiple submissions

^d^ Passive tick surveillance has been discontinued in some regions of Ontario and Québec; however, individuals could submit ticks directly to the National Microbiology Laboratory Branch

^e^ Passive tick surveillance has been discontinued in the entire province of Nova Scotia; however, individuals could submit ticks directly to the National Microbiology Laboratory Branch

Data on tick instar, level of engorgement and host were available for 99.9%, 0% and 100% of *I. pacificus*, respectively. Data on tick instar, level of engorgement and host were available for 99.4%, 99.3% and 99.6% of *I. scapularis*, respectively. Adult ticks were submitted most frequently, of which most were female (*I. scapularis*: 89.0%; *I. pacificus*: 93.8%) (**Table 2**). Larvae (0.3%; 0.4%) and nymphs (8.1%; 3.3%) were submitted less frequently. Overall, 44.0% of *I. scapularis* were partially or fully engorged. Humans were the most common host among *I. scapularis* and *I. pacificus* (90.3%, 94.4%, respectively), followed by dogs (7.7%, 5.4%, respectively).

**Table 2:** Instar, level of engorgement and host of *Ixodes scapularis* and *Ixodes pacificus* ticks submitted through passive surveillance, Canada, 2019^a,b^

Characteristic	Tick species			
	*Ixodes scapularis*		*Ixodes pacificus*	
	n	%	n	%
Instar				
Larva	27	0.3	3	0.4
Nymph	795	8.1	23	3.3
Adult female	8,719	89.0	647	93.8
Adult male	256	2.6	17	2.5
Total	9,797	100	690	100
Level of engorgement^c^				
Fully engorged	113	1.2	N/A	N/A
Partially engorged	4,188	42.8	N/A	N/A
Unfed	5,485	56.0	N/A	N/A
Total	9,786	100	N/A	N/A
Host				
Human	8,870	90.3	652	94.4
Dog	761	7.7	37	5.4
Cat	119	1.2	1	0.1
Other^d^	72	0.7	1	0.1
Total	9,822	100	691	100

Abbreviation: N/A, not applicable

^a^ Data are presented for all ticks where available, regardless of whether the tick was part of a single or a multiple submission

^b^ No passive surveillance was conducted in Yukon, Northwest Territories or Nunavut for *I. scapularis* or *I. pacificus* ticks. Passive tick surveillance has been discontinued in the entire province of Nova Scotia, and some regions of Ontario and Québec; however, individuals could submit ticks directly to the National Microbiology Laboratory Branch from these jurisdictions

^c^ Level of engorgement was not reported for *I. pacificus*

^d^ Includes environment, horse, rabbit, deer, skunk and other unspecified animal

**Figure 2:**
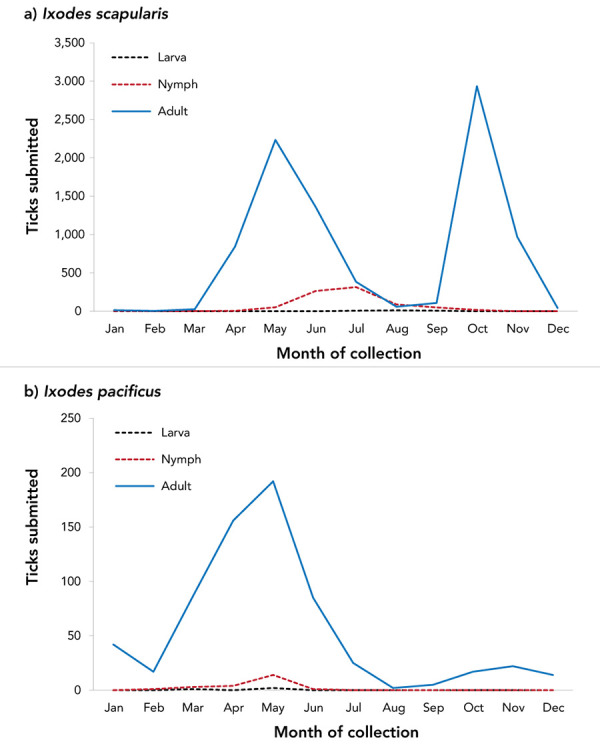
Number of *Ixodes scapularis* and *Ixodes pacificus* ticks submitted through passive surveillance, by month and tick instar, Canada, 2019^a,b^ ^a^ Data are presented for *I. scapularis* (n=9,797) and *I. pacificus* (n=690) ticks submitted through passive surveillance. Month of submission or tick instar was not available for *I. scapularis* (n=61) and *I. pacificus* (n=1) ^b^ No passive surveillance was conducted in Yukon, Northwest Territories or Nunavut for *I. scapularis* or *I. pacificus* ticks. Passive tick surveillance has been discontinued in the entire province of Nova Scotia, and some regions of Ontario and Québec; however, individuals could submit ticks directly to the National Microbiology Laboratory Branch from these jurisdictions

Month of acquisition was available for 99.9% of *I. pacificus* and 99.4% of *I. scapularis*. Locally acquired ticks were submitted in every month of the year (**Figure 2**). Submissions of *I. scapularis* adults peaked in May and October, while there was a single peak for *I. pacificus* adults in May. *Ixodes scapularis* nymph submissions peaked in June and July, while *I. pacificus* nymph submissions peaked in May.

### Passive surveillance infection prevalence

Data on laboratory testing were available for 97.4% of *I. pacificus* and 99.0%–99.5% of *I. scapularis*, depending on the pathogen. The most prevalent tick-borne pathogen was *B. burgdorferi*, found in 18.8% of *I. scapularis* (95% CI: 18.00–19.55), but only 0.3% of *I. pacificus* (95% CI: 0.05–0.97). Other tick-borne pathogens and co-infections were less prevalent (**Table 3**).

**Table 3:** Prevalence of *Borrelia burgdorferi*, *Anaplasma phagocytophilum* and *Babesia microti* infection in *Ixodes pacificus* and *Ixodes scapularis* ticks submitted through passive surveillance, Canada, 2019^a,b^

Pathogen	Infection prevalence			
	*Ixodes pacificus*		*Ixodes scapularis*	
Single agent	Maximum likelihood estimate^c^			
	%	95% CI	%	95% CI
*Borrelia burgdorferi*	0.3	0.05–0.97	18.8	18.00–19.55
*Anaplasma phagocytophilum*	N/A	N/A	1.4	1.22–1.70
*Babesia microti*	N/A	N/A	0.1	0.07–0.22
Any of above	0.3	0.05–0.97	20.0	19.23–20.83
Co-infection	Co-infection rate^d^			
	%	Number co-infected ticks/number ticks tested	%	Number co-infected ticks/number ticks tested
*Borrelia burgdorferi* + *Anaplasma phagocytophilum*	N/A	N/A	0.28	26/9,171
*Borrelia burgdorferi* + *Babesia microti*	N/A	N/A	0.02	2/9,171
*Anaplasma phagocytophilum* + *Babesia microti*	N/A	N/A	0.01	1/9,171
Any co-infection	N/A	N/A	0.32	29/9,171

Abbreviations: CI, confidence interval; N/A, not tested

^a^ All *I. pacificus* (n=691) and all *I. scapularis* from Alberta (n=55) were not tested for *A. phagocytophilum* and *B. microti*

^b^ No passive surveillance was conducted in Yukon, Northwest Territories or Nunavut for *I. scapularis* or *I. pacificus* ticks. Passive tick surveillance has been discontinued in the entire province of Nova Scotia, and some regions of Ontario and Québec; however, individuals could submit ticks directly to the National Microbiology Laboratory Branch from these jurisdictions

^c^ Maximum likelihood estimates of infection prevalence were used to account for pooled testing

^d^ Co-infection rate was calculated only among single submissions of ticks

Prevalence of *B. burgdorferi* was higher in *I. scapularis* from multiple submissions (24.5%, 95% CI: 20.64–28.69) than from single submissions (18.5%, 95% CI: 17.71–19.29) (**Table 4**). Prevalence did not significantly differ by submission type for any other pathogens.

**Table 4:** Prevalence of *Borrelia burgdorferi*, *Anaplasma phagocytophilum* and *Babesia microti* infections in *Ixodes scapularis* ticks submitted through passive surveillance by submission type and host, Canada, 2019^a^

Characteristic	Infection prevalence Maximum likelihood estimate							
	*Borrelia burgdorferi*		*Anaplasma phagocytophilum*		*Babesia microti*		Any of above	
	%	95% CI	%	95% CI	%	95% CI	%	95% CI
Submission type^b^								
Single	18.5	17.71–19.29	1.4	1.20–1.69	0.1	0.07–0.22	19.7	18.92–20.55
Multiple	24.5	20.64–28.69	1.7	0.89–3.06	0.2	0.01–0.82	26.3	22.31–30.70
Host^c^								
Human	19.2	18.39–20.04	1.3	1.11–1.59	0.1	0.07–0.23	20.4	19.54–21.23
Non-human^d^	14.7	12.44–17.13	2.6	1.68–3.85	0.1	0.01–0.57	16.7	14.31–19.29

Abbreviation: CI, confidence interval

^a^ No passive surveillance was conducted in Yukon, Northwest Territories or Nunavut for *I. scapularis* or *I. pacificus* ticks. Passive tick surveillance has been discontinued in the entire province of Nova Scotia, and some regions of Ontario and Québec; however, individuals could submit ticks directly to the National Microbiology Laboratory Branch from these jurisdictions

^b^ Single submissions consist of one tick. Multiple submissions consist of two or more ticks submitted together by the same person. All *I. scapularis* from Alberta were considered single submissions

^c^ Excludes *I. scapularis* where host is unknown or missing (n=43)

^d^ Non-human hosts include dog, cat, environment, horse, rabbit, deer, skunk or other unspecified animal

*Ixodes scapularis* submitted from human hosts had higher prevalence of *B. burgdorferi* infection (19.2%, 95% CI: 18.39–20.04) than those submitted from non-human hosts (14.7%, 95% CI: 12.44–17.13) (Table 4). However, *I. scapularis* submitted from non-human hosts had higher prevalence of *A. phagocytophilum* infection (2.6%, 95% CI: 1.68–3.85) than those submitted from human hosts (1.3%, 95% CI: 1.11–1.59). Both *B. burgdorferi*-infected *I. pacificus* ticks were from human hosts.

**Figure 3:**
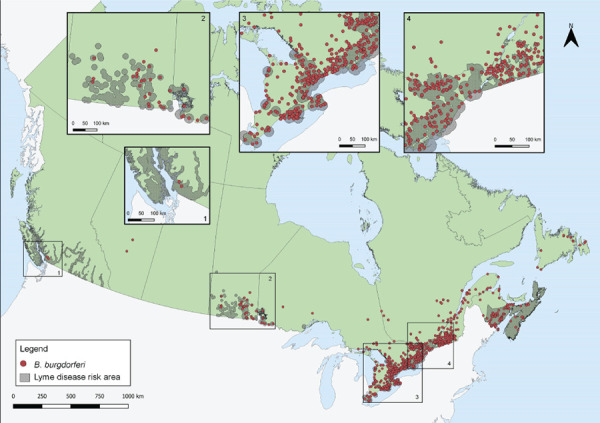
*Ixodes pacificus* and *Ixodes scapularis* ticks submitted through passive surveillance that were infected with *Borrelia burgdorferi*, Canada, 2019^a,b,c^ ^a^ Each dot represents the probable location of acquisition of at least one *I. pacificus* (n=2) or *I. scapularis* (n=1,819) single or multiple tick submission submitted through passive surveillance that was infected with *B. burgdorferi* ^b^ Lyme disease risk areas are identified by the provinces as of 2020 using the methods described in the 2016 national Lyme disease case definition (28)))) ^c^ No passive surveillance was conducted in Yukon, Northwest Territories or Nunavut for *I. scapularis* or *I. pacificus* ticks. Passive tick surveillance has been discontinued in the entire province of Nova Scotia, and some regions of Ontario and Québec; however, individuals could submit ticks directly to the National Microbiology Laboratory Branch from these jurisdictions

**Figure 4:**
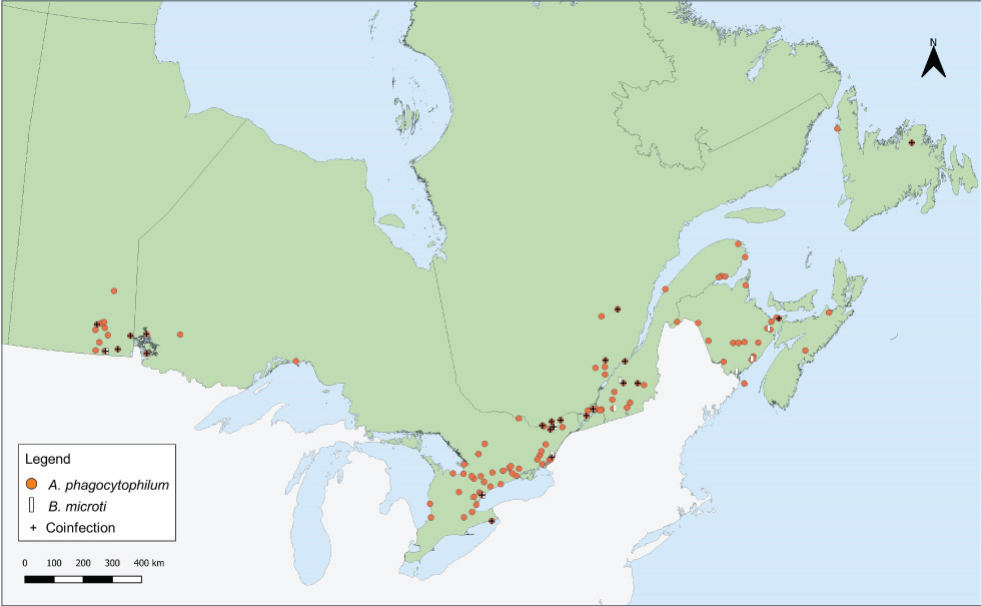
*Ixodes scapularis* ticks submitted through passive surveillance that were infected with *Anaplasma phagocytophilum*, *Babesia microti* and co-infections, Canada, 2019^a,b^ ^a^ Each symbol represents the probable location of acquisition of an *I. scapularis* single or multiple tick submission submitted through passive surveillance that tested positive for *A. phagocytophilum* (n=141), *B. microti* (n=13) or a co-infection (n=29). Co-infections were limited to only single submissions of *I. scapularis*, and include *B. burgdorferi* + *A. phagocytophilum* (n=26), *B. burgdorferi* + *B. microti* (n=2) and *A. phagocytophilum* + *B. microti* (n=1) ^b^ No passive surveillance was conducted in Yukon, Northwest Territories or Nunavut for *I. scapularis* or *I. pacificus* ticks. Passive tick surveillance has been discontinued in the entire province of Nova Scotia, and some regions of Ontario and Québec; however, individuals could submit ticks directly to the National Microbiology Laboratory Branch from these jurisdictions

Tick-borne pathogens were commonly found in ticks submitted from southern Manitoba, northwestern Ontario, southern and eastern Ontario, southern Québec and southern New Brunswick (**Figure 3** and **Figure 4**). Over two-thirds of *B. burgdorferi*-infected tick submissions were within previously identified LD risk areas (72.1%; n=1,313/1,821) (Figure 3). The majority of multiple submissions came from LD risk areas (76.9%; n=186/242), of which approximately half were infected with *B. burgdorferi* (51.4%; n=90/175). Newfoundland and Labrador, Nova Scotia and Québec all had higher infection prevalence of *B. burgdorferi* than the national average for *I. scapularis* (**Table 5**). Manitoba had the highest prevalence of *A. phagocytophilum* and *B. microti* infection among all provinces.

**Table 5:** Prevalence of *Borrelia burgdorferi*, *Anaplasma phagocytophilum* and *Babesia microti* infection in *Ixodes scapularis* and *Ixodes pacificus* ticks submitted through passive surveillance, by province, Canada, 2019^a^

Province	Infection prevalence Maximum likelihood estimate					
	*Borrelia burgdorferi*		*Anaplasma phagocytophilum*		*Babesia microti*	
	%	95% CI	%	95% CI	%	95% CI
*Ixodes pacificus*						
British Columbia	0.3	0.05–0.97	N/A	N/A	N/A	N/A
*Ixodes scapularis*						
Alberta^b^	5.5	1.45–14.01	N/A	N/A	N/A	N/A
Saskatchewan	0.0	0–56.15	0.0	0–56.15	0.0	0–56.15
Manitoba	18.3	12.94–24.68	10.4	6.38–15.81	2.4	0.78–5.63
Ontario	18.3	17.37–19.22	0.9	0.73–1.18	0.1	0.02–0.14
Québec	24.2	22.18–26.30	1.9	1.32–2.63	0.1	0.02–0.39
Newfoundland and Labrador	29.5	17.63–44.01	4.6	0.82–14.28	0.0	0–8.02
New Brunswick	12.8	10.80–15.10	2.6	1.70–3.74	0.3	0.08–0.87
Nova Scotia	26.2	15.38–39.82	3.9	0.70–12.31	0.0	0–6.82
Prince Edward Island	0.0	0–21.53	0.0	0–21.53	0.0	0–21.53
Total	18.8	18.00–19.55	1.5	1.22–1.70	0.1	0.07–0.22

Abbreviations: CI, confidence interval; N/A, not tested

^a^ No passive surveillance was conducted in Yukon, Northwest Territories or Nunavut for *I. scapularis* or *I. pacificus* ticks. Passive tick surveillance has been discontinued in the entire province of Nova Scotia, and some regions of Ontario and Québec; however, individuals could submit ticks directly to the National Microbiology Laboratory Branch from these jurisdictions

^b^ Excludes *I. pacificus* found in the province (n=1) which tested negative for *B. burgdorferi*

### Active surveillance tick characteristics

In active surveillance, *I. scapularis* and *I. pacificus* were found at 78 of 184 surveillance sites (range of ticks found: n=0–130). *Ixodes scapularis* (n=1,156) were found in Manitoba, Ontario, Québec, New Brunswick, Nova Scotia and Prince Edward Island, while *I. pacificus* (n=10) were found in British Columbia. Regarding the instar, 51.5% (n=601/1,166) of ticks were identified as nymphs, 29.5% (n=344/1,166) were adults and 19.0% (n=221/1,166) were larvae.

### Active surveillance infection prevalence

**Figure 5:**
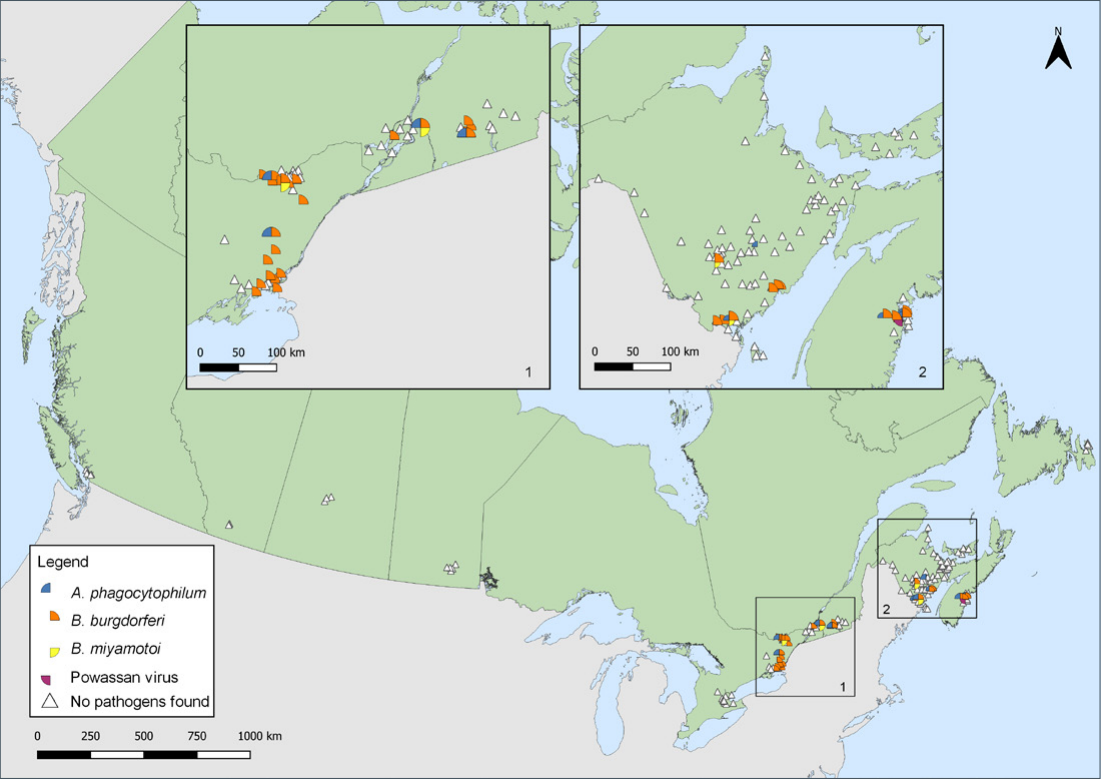
*Ixodes scapularis* and *Ixodes pacificus* ticks with associated pathogens collected through active surveillance, Canada, 2019^a,b^ ^a^ Each symbol represents an active surveillance site where *B. burgdorferi* (n=38), *A. phagocytophilum* (n=12), *B. miyamotoi* (n=4), or Powassan virus (n=1) were found in *I. scapularis* ticks. There were 142 sites where no tick-borne pathogens were identified in ticks, including sites where no *I. scapularis* or *I. pacificus* were found (n=108). No active surveillance was conducted in Yukon, Northwest Territories or Nunavut for *I. scapularis* or *I. pacificus* ticks. The presence or absence of infected *I. scapularis* ticks is influenced by varying level of effort of active surveillance between provinces and seasonal variation when active surveillance took place. Infection prevalence should be interpreted with caution as not all active surveillance conducted in 2019 in Canada is included ^b^ Number of ticks tested: British Columbia (n=10), Alberta n=(0), Saskatchewan (n=0), Manitoba (n=3), Ontario (n=188–406 depending on pathogen), Québec (n=141), Newfoundland and Labrador (n=0), New Brunswick (n=194), Nova Scotia (n=169) and Prince Edward Island (n=2)

Data on laboratory testing were available for 100% of *I. pacificus* collected and 73.8%–98.3% of *I. scapularis* nymphs and adults collected, depending on the pathogen. No tick-borne pathogens were found in *I. pacificus* (**Table 6**). In *I. scapularis*, *B. burgdorferi* was identified in 26.2% of ticks tested and in four provinces: Ontario, Québec, New Brunswick, and Nova Scotia. *Anaplasma phagocytophilum* was identified in the same four provinces in 3.4% of *I. scapularis*. *Borrelia miyamotoi* and POWV were found in 0.5% or fewer. **Figure 5** shows the locations of ticks with tick-borne pathogens collected in active surveillance.

**Table 6:** Infection prevalence of *Ixodes scapularis* and *Ixodes pacificus* ticks collected in active surveillance, by province, Canada, 2019^a,b^

Province	Infection prevalence									
	*Anaplasma phagocytophilum*		*Babesia microti*		*Borrelia burgdorferi*		*Borrelia miyamotoi*		Powassan virus	
	Number positive tick/number tick tested	%	Number positive tick/number tick tested	%	Number positive tick/number tick tested	%	Number positive tick/number tick tested	%	Number positive tick/number tick tested	%
*Ixodes pacificus*										
British Columbia	0/10	0	0/10	0	0/10	0	0/10	0	0/10	0
*Ixodes scapularis*										
Manitoba	0/3	0	0/3	0	0/3	0	0/3	0	0/3	0
Ontario	14/406	3.5	0/397	0	126/410	30.7	1/410	0.2	0/188	0
Québec	2/141	1.4	0/141	0	28/141	19.8	1/141	0.7	0/141	0
New Brunswick	8/194	4.1	0/194	0	41/194	21.1	3/194	1.6	0/194	0
Nova Scotia	7/169	4.1	0/169	0	46/169	27.2	0/169	0	1/169	0.6
Prince Edward Island	0/2	0	0/2	0	0/2	0	0/2	0	0/2	0
Total	31/915	3.4	0/906	0	241/919	26.2	5/919	0.5	1/697	0.1

^a^ No *I. scapularis* or *I. pacificus* ticks were collected or tested in Alberta, Saskatchewan or Newfoundland and Labrador through active surveillance. No active surveillance was conducted in Yukon, Northwest Territories or Nunavut for *I. scapularis* or *I. pacificus* ticks

^b^ Infection prevalence is influenced by varying level of effort of active surveillance between provinces and seasonal variation when active surveillance took place. Infection prevalence should be interpreted with caution as not all active surveillance conducted in 2019 in Canada is included

## Discussion

In 2019, 9,858 *I. scapularis* and 691 *I. pacificus* were submitted in passive surveillance in eight provinces. Of these, 20.0% of *I. scapularis* and 0.3% of *I. pacificus* were infected with at least one of the tick-borne pathogens tested, including *B. burgdorferi*, *A. phagocytophilum* or *B. microti*. Active surveillance identified four tick-borne pathogens among *I. scapularis* collected in four provinces, and no tick-borne pathogens among the few *I. pacificus* collected.

In passive surveillance, one *I. pacificus* with no travel history was identified in Alberta, outside of British Columbia where reproducing populations are known to be established. *Ixodes pacificus* have been found in the province before on migratory birds ( ( ((29)))), or from human or animal hosts mostly associated with travel ( ( ((20)))).

Ticks were submitted through passive surveillance in every month, highlighting the potential year-round risk (depending on location and weather) of exposure to ticks, which may or may not be infected with tick-borne pathogen(s). *Ixodes* spp. ticks, for example, were often found in western Canada in the winter but were rarely infected ( ( ((23)))). The single peak of *I. pacificus* tick submissions in the spring has historically been observed in British Columbia ( ( ((23)))) and in the western United States ( ( ((30)))), as nymphs and adults are both active during the cooler spring months ( ( ((31)))). Bimodal peaks for adult *I. scapularis* in late spring and autumn have been previously observed in central and eastern Canada ( ( ((3,16,32)))), and are consistent with adult *I. scapularis* activity in a 3 to 4-year lifecycle extended, in part, by cooler spring temperatures ( ( ((31,33)))). Nymphs of both species, which are most implicated in LD transmission ( ( ((34)))), peak during the late spring to summer months when LD onset in humans also peaks ( ( ((9)))).

Compared to most recent estimates, infection prevalence of *B. burgdorferi* among *I. pacificus* ticks in British Columbia (0.3%) was consistent with annual rates from 2002 to 2018 between 0.1 and 0.4% ( ( ((23)))). In Manitoba, infection prevalence among *I. scapularis* was lower (18.3%) than the 2018 minimum infection rate of 20.7% ( ( ((35)))). In Ontario, infection prevalence among *I. scapularis* increased to 18.3% from the 2011–2017 rate of 15.8% ( ( ((36)))). In Québec, infection prevalence also increased to 24.2% from 17.6% in *I. scapularis* adults from 2009 to 2015 ( ( ((37)))). Inter and intra-provincial variability in annual prevalence is influenced, however, by annual variation in weather, effort of surveillance, history of established vector populations and habitat suitability.

Prevalence of *I. scapularis* being infected with at least one of the tick-borne pathogens tested was higher in multiple submissions than single submissions. As multiple submissions are indicators of tick establishment in a given area ( ( ((38)))), this suggests higher infection prevalence among established tick populations.

Over two-thirds of *B. burgdorferi*-infected ticks had probable locations of acquisition within LD risk areas. The LD risk areas are identified by the provinces using the methods described in the 2016 national LD case definition ( ( ((28)))) and are regularly updated to incorporate new surveillance data. *Borrelia burgdorferi*-infected ticks collected outside of these known LD risk areas may be adventitious ticks, brought to these areas by migratory birds or terrestrial hosts ( ( ((18)))). Public health authorities and clinicians should be aware that risk of exposure to infected ticks exists outside of known LD risk areas. Increasing the collaborative effort of active surveillance can support the timely recognition of new LD risk areas. Promptly identifying and removing ticks, regardless of their locality of acquisition, can prevent transmission of tick-borne pathogens.

In active surveillance, there was geographic variability in infection prevalence, similar to findings from passive surveillance. Conducting standardized and consistent active surveillance across the country can help identify new LD-risk areas and detect other emerging tick-borne pathogens in known LD-risk areas, thereby informing local risk of exposure to TBD.

While *B. burgdorferi* was the most prevalent tick-borne pathogen in both passive and active surveillance, *A. phagocytophilum*, *B. microti*, *B. miyamotoi* and POWV were also detected. All provinces, however, had lower infection prevalence than hyper-endemic areas in the northeastern United States. For example, *I. scapularis* adults collected in Maine through passive surveillance had *B. burgdorferi*, *A. phagocytophilum* and *B. microti* infection prevalence of 42.4%, 11.1% and 6.5%, respectively ( ( ((39)))).

Ongoing climate and environmental changes affect TBD risk in a variety of ways, by altering populations of ticks and their animal hosts, as well as increasing human exposure to ticks ( ( ((1)))). As current projections predict an increased risk of TBD from expansion of *Ixodes* spp. habitat in the future ( ( ((1,5,40)))), continued surveillance can monitor changes in tick distribution and infection prevalence. More studies are also needed to understand the emergence and ecology of other tick-borne pathogens across Canada, which may differ from *B. burgdorferi*, for example, in their enzootic transmission cycles ( ( ((41)))).

### Strengths and limitations

This inaugural article combining active and passive tick surveillance presents a national snapshot of tick vectors and their emerging associated pathogens. By integrating the two types of surveillance, the strengths and weaknesses of the individual systems are complemented. Whereas active surveillance is resource-intensive and therefore limited in geographic scope, passive surveillance programs can be implemented on a larger geographic scale; however, passive surveillance lacks specificity as it often collects adventitious ticks seeded by migratory birds, especially ticks collected from companion animal hosts which readily acquire ticks from the environment ( ( ((18,38)))).

There are several limitations to this study. Provincial passive surveillance programs, and the effort and timing of active surveillance, vary across Canada due to resource limitations or logistics. Passive tick surveillance has been discontinued or limited to specific hosts in several regions. Further, passive tick surveillance can be limited by public awareness, and geographic or host-specific biases in tick submissions ( ( ((3,42,43)))). Not all active surveillance conducted in Canada in 2019 was included in this study; data from the many groups that conduct active surveillance, which includes university researchers, Indigenous communities and local or provincial public health units, was not all available. These limitations lead to underestimating the number of ticks, which affects the accuracy of infection prevalence. Lastly, it may be inappropriate to pool data from multiple active and passive surveillance systems due to differences in methodology between sources.

## Conclusion

Passive and active surveillance identified both *I. scapularis* and *I. pacificus* across Canada in varying amounts depending on location, including some ticks which were infected with tick-borne pathogen(s). Both passive and active tick surveillance have utility in signalling and confirming new LD risk areas, which can be used to inform public health authorities where environmental risk for LD occurs. This information is used to communicate the local risk of LD and TBD to the public as well as to healthcare workers. Continued surveillance will be crucial for monitoring any expansion of areas at risk of exposure to ticks and tick-borne pathogens, and to appropriately target public health interventions such as education and awareness campaigns towards at-risk areas.
